# Identification and validation of ubiquitination-associated genes of senile osteoporosis based on bioinformatics analysis

**DOI:** 10.3389/fimmu.2025.1629276

**Published:** 2025-12-12

**Authors:** Xiyue Cheng, Junchuan Liu, Yiman Guan, Boya Jing, Jing Zhao, Yan Cao

**Affiliations:** 1 Department of General Practice, Hebei Medical University Third Hospital, Shijiazhuang, China; 2 First Department of Comprehensive Orthopedics, Hebei Medical University Third Hospital, Shijiazhuang, China; 3 Department of Ultrasound, Hebei Medical University Third Hospital, Shijiazhuang, China

**Keywords:** senile osteoporosis, ubiquitination, biomarkers, diagnosis, RPS27a, Ube2E1

## Abstract

**Background:**

Senile osteoporosis (SOP) is linked to the ubiquitination process, with dysregulation of ubiquitin-mediated protein turnover disrupting bone remodeling and resulting in decreased bone mineral density (BMD). This study aimed to identify biomarkers related to ubiquitination in SOP and explore their molecular regulatory mechanisms.

**Methods:**

Transcriptomic data of SOP samples (categorized by high and low BMD) were obtained from public databases. Differential expression analysis, protein-protein interaction networks, and the CytoHubba plugin (using Maximum Neighborhood Component and degree algorithms) were utilized, alongside the Least Absolute Shrinkage and Selection Operator, to identify ubiquitination-related genes (URGs) as potential SOP biomarkers. The diagnostic potential of these biomarkers was assessed through a Support Vector Machine model and a nomogram. Their molecular mechanisms were further investigated using enrichment analysis, immune infiltration analysis, and the construction of regulatory networks. Expression levels of the biomarkers were validated in a SOP rat model, with enzyme-linked immunosorbent assay applied to detect relevant indices.

**Results:**

RPS27A and UBE2E1 were significantly underexpressed in low BMD samples and demonstrated a strong ability to differentiate between patients with varying BMDs, making them potential diagnostic biomarkers for SOP. A positive correlation was observed between RPS27A and UBE2E1 (cor = 0.35, *P* = 0.026). Both genes were involved in neurodegenerative diseases, critical cellular functions, and key intracellular signaling pathways. Additionally, RPS27A showed a positive correlation with macrophages and monocytes, whereas UBE2E1 exhibited a negative correlation with T follicular helper cells (Tfh) and T helper 17 cells (Th17). The transcription factor MAX and miRNA hsa-miR-106b-5p were identified as potential regulators of both biomarkers. Western blot, immunohistochemistry, and reverse transcription quantitative PCR further confirmed significantly lower expression of RPS27A and UBE2E1 in the SOP group compared to the Sham group.

**Conclusion:**

This study successfully identified RPS27A and UBE2E1 as key biomarkers for SOP, demonstrating their diagnostic potential and involvement in important biological pathways and immune responses, thus offering new prospects for therapeutic interventions.

## 1 Introduction

Osteoporosis (OP) is a chronic metabolic bone disorder that poses a significant risk of fractures and mortality in the elderly. It is typically classified into primary and secondary forms (1). Senile OP (SOP), a subtype of primary OP, is primarily caused by age-related abnormalities in bone metabolism in individuals over the age of 70 (2). The pathophysiology in older women is often linked to postmenopausal estrogen deficiency and the natural aging process, leading to the perception that the development of primary OP, particularly in older women, is inevitable (3). The pathogenesis of OP in elderly women is multifactorial. In addition to aging and estrogen deficiency, factors such as poor diet, physical inactivity, and insufficient sunlight exposure can also contribute to reduced bone mass and mineral density (4). Bone mineral density (BMD) is the most commonly used indicator of bone health, encompassing the structural integrity, metabolic transformation, cumulative bone damage (microfractures), and mineralization of bone tissue (5). BMD measurement provides a means to assess bone health status and predict the degree of OP, which is also critical for assessing fracture risk (6). While BMD remains the gold standard for diagnosing OP, its diagnostic utility is not without limitations (7). In recent years, researchers have sought to explore the mechanisms underlying SOP from perspectives such as immune system dysfunction, mitochondrial impairment, and genetic predisposition, aiming to identify novel diagnostic approaches (8–10). Therefore, the identification of new and effective biomarkers for the early diagnosis and prevention of SOP is of paramount importance.

Ubiquitination is a key post-translational modification of proteins and represents the most significant and effective protein degradation pathway in mammalian cells (11). The ubiquitination process involves three key enzymes: ubiquitin-activating enzyme (E1), ubiquitin-conjugating enzyme (E2), and ubiquitin protein ligase (E3). This process plays a critical role in the degradation of short-lived proteins and the regulation of essential biological functions, including cell proliferation, differentiation, and the growth and development of tissues and organs (12–14). Recent research has increasingly highlighted the significant role of ubiquitination in bone remodeling. OP is associated with the abnormal expression and dysfunction of ubiquitination and de-ubiquitination enzymes (15). For instance, the ubiquitin ligase Smurf1 can ubiquitinate downstream BMP molecules, leading to their degradation and inhibition of osteoblast differentiation (16, 17). Additionally, studies have shown that the ubiquitin-binding enzyme UBE2E3 influences the senescence and osteogenic differentiation of bone marrow stromal cells (BMSCs) (18). Conversely, deubiquitinating enzymes remove ubiquitin from substrate proteins, thereby modulating bone remodeling (19, 20). Hence, ubiquitin plays a pivotal role in the onset and progression of OP in the elderly, warranting further investigation into its specific mechanisms in bone reconstruction.

This study was based on the transcriptome data of “elderly postmenopausal women with different bone densities” from public databases. Through bioinformatics analysis, the ubiquitination-related genes RPS27A and UBE2E1 were selected as potential key biomarkers. The study systematically explored the diagnostic value and molecular regulatory mechanisms of these two genes in postmenopausal osteoporosis, and verified their expression levels and correlations with bone metabolism indicators in a model of elderly postmenopausal rats. To date, no research has adopted a combined strategy of bioinformatics and experimental validation to jointly identify RPS27A and UBE2E1 as biomarkers related to ubiquitination in elderly osteoporosis. Therefore, this study provides new theoretical basis for the early diagnosis, prevention strategies, and targeted treatment development of this disease.

## 2 Methods

### 2.1 Data source

Transcriptome datasets were sourced from the Gene Expression Omnibus (GEO) database (https://www.ncbi.nlm.nih.gov/gds). The GSE13850 dataset was derived from the GPL96 platform. It contained B-cell samples from the blood of 20 elderly postmenopausal women with osteoporosis, as well as B-cell samples from the blood of 20 age-matched controls. This dataset was used as the training cohort. The GSE56815 dataset (21) was also based on the GPL96 platform. It included monocyte samples from the blood of 20 postmenopausal women with osteoporosis and monocyte samples from the blood of 20 controls. All post-surgical samples were excluded in the study, and the dataset was ultimately used as the validation cohort. The miRNA sequencing dataset GSE64433 was derived from the GPL18402 platform. It contained whole blood samples from 3 patients with osteoporosis (OP) and 3 controls, and was mainly used for the construction of the regulatory network in this study. The URGs utilized in this study were downloaded from the Integrated Ubiquitination Site and Ubiquitin-conjugating Enzyme Database (iUUCD) (http://iuucd.biocuckoo.org/), initially containing 1,394 genes. After removing duplicates and replacing aliases with gene symbols, 1,367 unique genes were retained.

### 2.2 Determination of differentially expressed URGs

Differentially expressed genes (DEGs) between high and low BMD samples in the GSE13850 dataset were identified using the limma package (v3.58.1) (22). The screening threshold was set as p-value < 0.05 and |log_2_ fold change (log_2_FC)| > 0, and the selection of this threshold was based on the literature (23). The resulting DEGs were visualized as a volcano plot using the ggplot2 package (v3.3.6) (24) and as a heatmap using the ComplexHeatmap package (v2.4.2) (25). Subsequently, intersections between these DEGs and the 1,367 URGs were determined using the ggVenndiagram package (v1.2.3) (26), generating a set of DE-URGs. Enrichment analyses of the DE-URGs, including Gene Ontology (GO) (*P* < 0.05) and Kyoto Encyclopedia of Genes and Genomes (KEGG) (*P* < 0.05), were conducted using the clusterProfiler package (v3.18.1) (27) to explore their biological functions and associated pathways.

### 2.3 Selection of candidate genes

To investigate potential protein-level interactions among DE-URGs, these genes were imported into the STRING database (http://string-db.org) to build a protein-protein interaction (PPI) network (highest confidence = 0.4). The resulting PPI network was visualized using Cytoscape software (v3.10.0) (28). The CytoHubba plugin within Cytoscape was then used, applying two algorithms—Degree and Maximal Clique Centrality (MCC)—to identify the top 10 influential genes from each algorithm. These top 10 genes were analyzed for intersection points, ultimately screening for candidate genes relevant to this study.

### 2.4 Recognition of biomarkers and construction of nomogram

The glmnet package (v 4.1-8) (29) was used to construct a least absolute shrinkage and selection operator (LASSO) regression model based on the candidate genes, with specific parameters set as follows: the penalty type was set as L1 regularization, and 5-fold cross-validation was adopted; during each iteration, the data was randomly divided into a training set (4/5) and a validation set (1/5) to select the optimal lambda value (lambda.min) that minimized the prediction error. Based on this optimal lambda value, feature genes corresponding to non-zero coefficients were extracted to further identify candidate genes that made significant contributions to the binary outcome. Afterwards, based on the genes screened by LASSO, the e1071 package (23) was used to construct an Support Vector Machine (SVM) model, with parameters set as follows: the radial basis kernel was selected as the kernel function (‘kernel=“radial”‘); the regularization parameter C was set to 1; and the kernel function parameter gamma was set to ‘scale=TRUE’. The diagnostic performance of the model was evaluated by plotting a receiver operating characteristic (ROC) curve and calculating the area under the curve (AUC). The performance of the classification model was further assessed by constructing a Precision-Recall (PR) curve to examine the relationship between precision and recall across varying thresholds. A decision curve was applied to assess the model’s net benefit. Additionally, the expression of these genes was analyzed within both training and validation cohorts, comparing samples with high and low BMD. Outcomes were visualized using box plots created with the ggplot2 package. Genes exhibiting significant differences and consistent expression patterns across both datasets were selected as biomarkers (*P* < 0.05).

Subsequently, the rms package (v 6.5-0) (30) was used to integrate the identified biomarkers and develop a nomogram model within the training cohort. Each biomarker was assigned a specific score, with the cumulative score representing the total score. This total score was then used to estimate the probability of developing SOP, with higher scores indicating a greater likelihood of SOP occurrence. A calibration curve was plotted to evaluate the model’s predictive accuracy, and a decision curve was used to assess its clinical utility. Additionally, a clinical impact curve was generated to analyze the clinical response rate of the model.

### 2.5 Functional and annotation analysis

Following biomarker identification, a comprehensive analysis was conducted to elucidate their intrinsic functions. In the training cohort, correlations between biomarkers were assessed based on their expression levels using the corrplot package (v0.92) (31) with the Spearman method. Gene Set Enrichment Analysis (GSEA) was then performed using the clusterProfiler package, applied to the expression matrix sorted by correlation (*P* < 0.05). The reference gene set used was c2.cp.kegg_legacy.v2023.2.Hs.symbols.gmt, sourced from the Molecular Signatures Database (MSigDB) (https://www.gsea-msigdb.org/gsea/msigdb/). To explore the subcellular localizations of the biomarkers, each was queried in the GeneCards database (https://www.genecards.org/), focusing on entries with the highest confidence level (Confidence = 5) to determine the subcellular localization of the proteins encoded by these genes.

### 2.6 Immune infiltration analysis

To further evaluate the immune microenvironment in SOP patients with varying BMD, immune infiltration assays were conducted. The ImmuCellAI algorithm (32) was applied to quantify the abundance of 25 distinct immune cells in the OP and control samples from the GSE13850 dataset. Spearman correlation analysis was then applied to explore the relationships between biomarkers and immune cell variations across groups (|cor| > 0.3 and *P* < 0.05). This integrated approach provided insights into the immune interactions associated with SOP.

### 2.7 Disease association analysis and drug prediction studies

To assess the relationship between biomarkers and SOP, this study further evaluated their associations using the Comparative Toxicogenomics Database (CTD) (http://ctdbase.org), utilizing the inference score to measure the strength of these associations. In the search for potential therapeutic drugs for SOP, DGIdb (https://www.dgidb.org/) was queried for drugs targeting the identified biomarkers, and a network diagram was constructed for visualization.

### 2.8 Construction of regulatory network

To elucidate the molecular regulatory mechanisms of the biomarkers, regulatory networks were constructed. First, using the miRNet database (https://www.mirnet.ca) with TRRUST parameters, transcription factors (TFs) interacting with the biomarkers were predicted based on the training cohort. Differential analysis was then performed with the limma package on the GSE64433 dataset, which contained samples from three OP and three control subjects, to identify differentially expressed miRNAs (DE-miRNAs) [|log_2_Fold Change (FC)| > 1 and *P* < 0.05]. The multiMiR package (v3.19) (33) was employed to identify miRNAs potentially regulating the biomarkers (‘table = “validated”, ‘org = “hsa”). The intersection of DE-miRNAs and predicted miRNAs with opposite expression to the biomarkers generated the terminal miRNAs, forming a miRNA-mRNA regulatory network. Additionally, lncRNAs regulating these miRNAs were predicted using the starBase database (https://rnasysu.com/encori/), leading to the construction of an lncRNA-miRNA-mRNA network. All networks were visualized using Cytoscape software.

### 2.9 Animal model construction and sample collection

Six female Sprague-Dawley (SD) rats, aged 8 weeks, were purchased from Beijing Huafukang Biotechnology Co., Ltd. (Production Permit No.: SCXK (Jing) 2024-0003, Usage Permit No.: SYXK (Dian) K2022-0007). After one week of adaptive feeding, the rats were randomly and evenly assigned to the Sham group (n=3) and the SOP group (n=3). The rats were housed under controlled temperature conditions, with free access to food and water. D-galactose was injected into the nape of the rats’ necks for 45 consecutive days to induce senescence. Following this, ovariectomy was performed to establish the SOP model. Body weights of the rats were recorded weekly. After 14 weeks, all rats were anesthetized, and blood was collected from the heart. The blood was allowed to stand at room temperature for 2 hours before centrifugation to obtain serum samples for enzyme-linked immunosorbent assay (ELISA) testing. Subsequently, the rats were euthanized, and their left femurs were collected for Western blot (WB) analysis and reverse transcription quantitative PCR (RT-qPCR) analysis. All procedures were approved by the Animal Ethics Committee of Yunnan Bestime Biological Technology LLC.

### 2.10 Hematoxylin-eosin staining and immunohistochemicalanalysis

The right femoral tissue was removed and fixed in 4% paraformaldehyde. After trimming and fixing, the tissue was dehydrated, infiltrated with wax, and embedded in paraffin. Sections were cut and mounted on slides for further analysis. H&E staining was performed to observe the pathological changes in the femoral tissue of the SD rats. IHC analysis was conducted to detect the expression of biomarkers in the femoral tissue. Each experimental technique was repeated three times. Images of the sections were captured using an inverted microscope (Nikon, TS2), and the results were analyzed using ImageJ Pro-Plus software. Statistical analysis was performed using GraphPad Prism 5 software, and intergroup differences were compared by t-test. A P-value < 0.05 was considered statistically significant.

### 2.11 ELISA analysis

To assess the occurrence of OP, an ELISA detection kit was used to measure the levels of osteocalcin (BGP) (Meimian: LY3497-B, China), tartrate-resistant acid phosphatase 5b (TRACP5b) (Meimian: LY3282-B, China), and osteoprotegerin (OPG) (Meimian: LY2991-B, China) in the rats’ serum. The procedure was followed according to the manufacturer’s instructions. Optical density (OD) values were measured at 450 nm using a microplate reader (BioTek: EIx800, USA) within 15 minutes. Experimental technique was repeated three times. Statistical analysis was performed using GraphPad Prism 5 software, and intergroup differences were compared by t-test. A P-value < 0.05 was considered statistically significant.

### 2.12 WB analysis

Proteins were extracted from the left femoral tissue using RIPA lysis buffer (Servicebio, Servicebio), and protein concentrations were measured using a BCA protein quantification kit (Beyotime, P0009). The appropriate amount of 5× protein loading buffer (Servicebio, G2013-100ML) was then added to the proteins, and the mixture was heated in a boiling water bath for 10 minutes to denature the proteins. SDS-PAGE electrophoresis was performed to separate the protein compounds, which were then transferred onto a PVDF membrane (Millipore, K2MA8350E). The membrane was blocked and incubated with the corresponding primary antibody solution overnight at 4 °C. On the following day, the membrane was incubated with the secondary antibody at room temperature for 60 minutes. The antibody dilution ratios are detailed in **Supplementary Table S1**. The membrane was exposed using an ECL chemiluminescent substrate (Affinity, KF8001) on a gel imaging system, and the gray values were analyzed using ImageJ software. Experimental technique was repeated three times. Statistical analysis was performed using GraphPad Prism 5 software, and intergroup differences were compared by t-test. A P-value < 0.05 was considered statistically significant.

### 2.13 RT-qPCR analysis

Left femur tissue samples from 3 Sham and 3 SOP rats were collected. Total RNA was extracted from these samples using TRIzol reagent (Ambion, USA). mRNA was reverse-transcribed into cDNA using a test kit (Yeasen). The cDNA was diluted with RNase/DNase-free reagents. The specific reaction system and primer sequences are listed in **Supplementary Table S2**. RT-qPCR analysis was then performed according to the amplification conditions shown in **Supplementary Table S2**. The data were analyzed using the 2^–ΔΔCt^ method, with GAPDH as the reference gene for normalization (34). Experimental technique was repeated three times. Statistical analysis was performed using GraphPad Prism 5 software, and intergroup differences were compared by t-test. A P-value < 0.05 was considered statistically significant.

### 2.14 Statistical analysis

All analyses were performed using R software (v4.2.2). Differences between groups were assessed using the Wilcoxon test. For RT-qPCR, WB, ELISA, and IHC, comparisons of 2^–ΔΔCt^ values were performed using the t-test (*P* < 0.05).

## 3 Results

### 3.1 Identification of 817 DEGs and 76 DE-URGs

**Figure 1:**
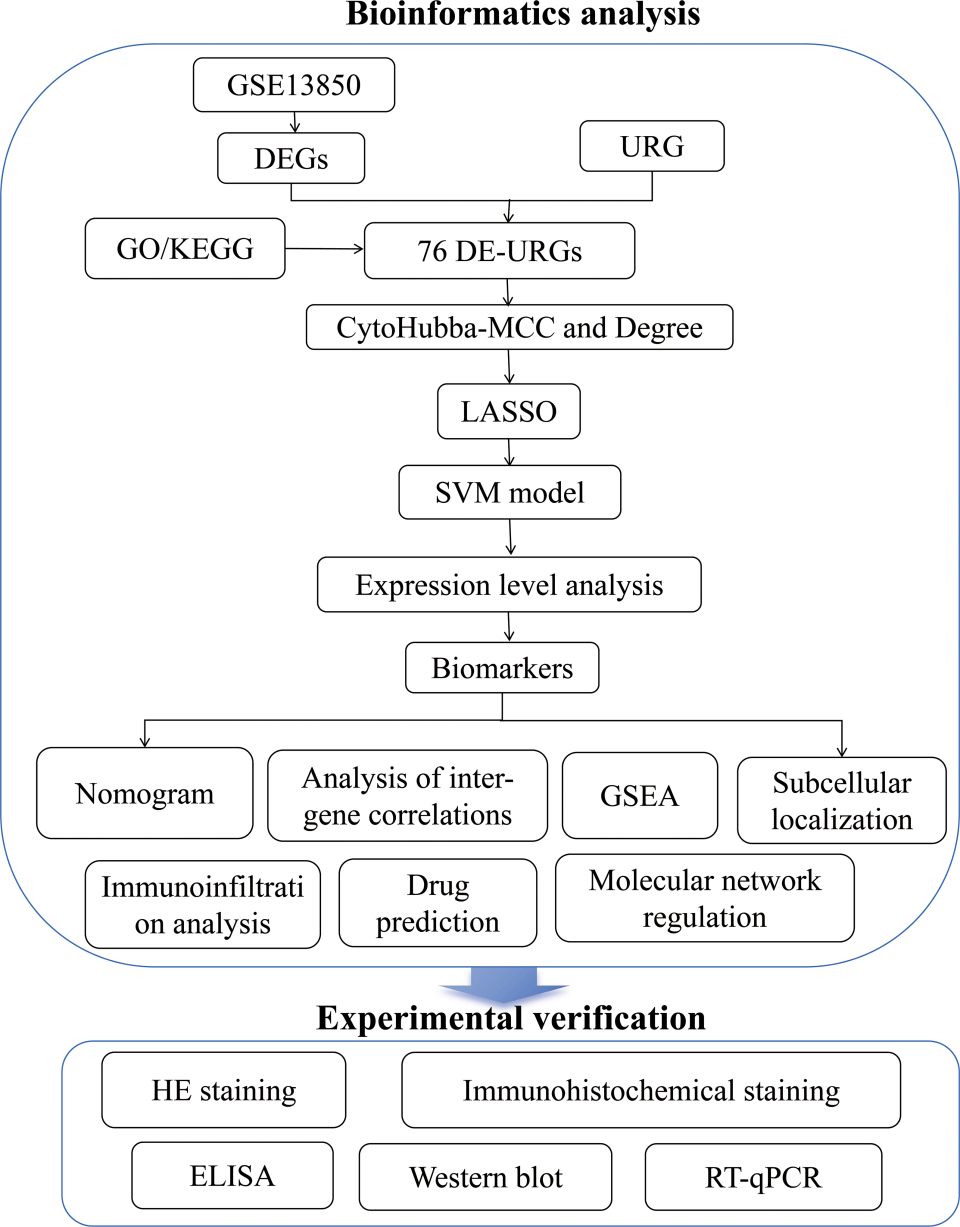
Flow chart.

**Figure 2:**
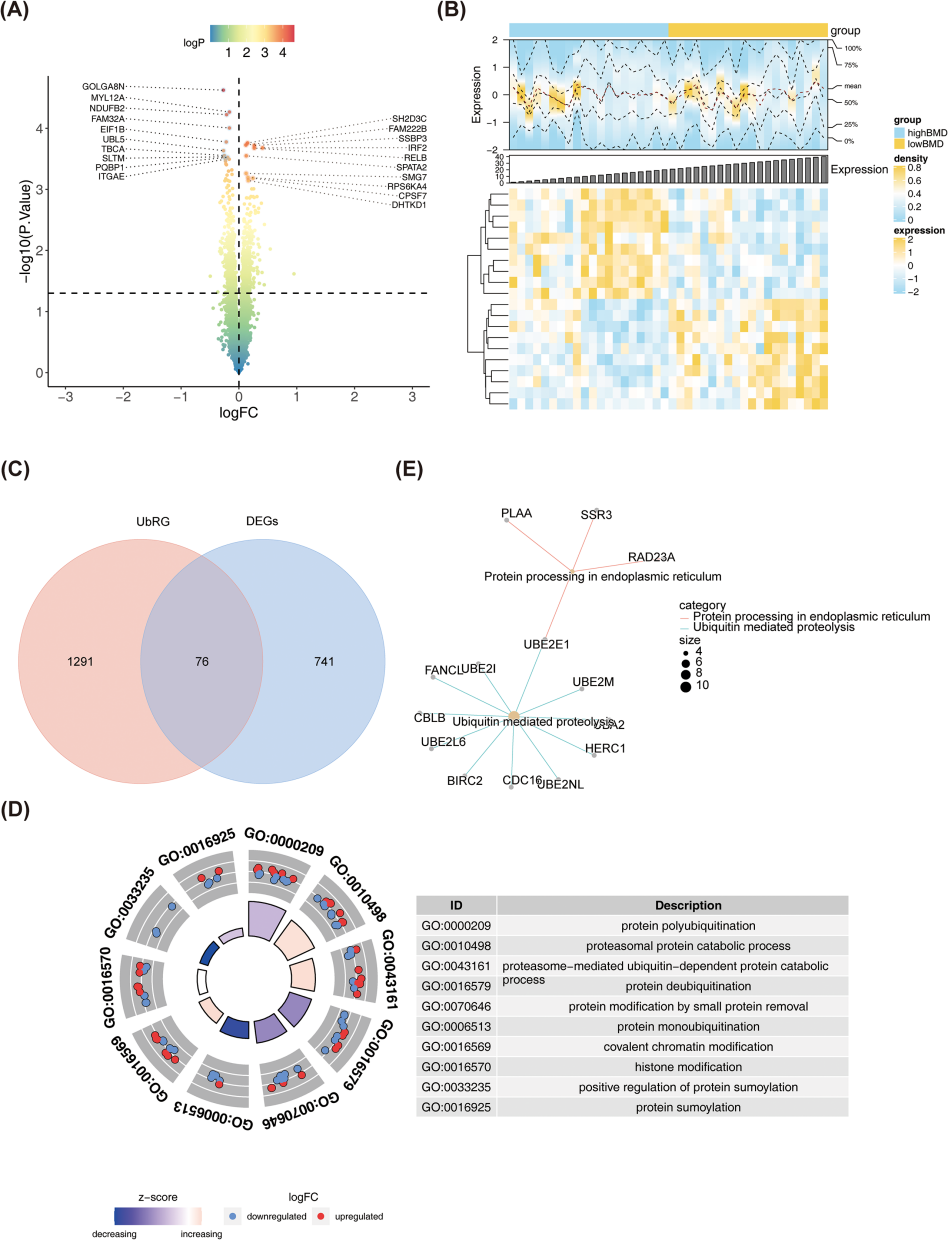
Identification of differentially expressed genes and ubiquitination-related genes in low bone mineral density samples. **(A)** Volcano map of differentially expressed genes in high and low bone mineral density samples. Divided by reference lines, the genes in the upper right corner are upregulated genes, the genes in the upper left corner are downregulated genes, and the rest are genes with no significant statistical difference. **(B)** Heat maps of differentially expressed genes in high and low bone mineral density samples. Yellow indicates highly expressed genes, and blue indicates lowly expressed genes. **(C)** Venn diagram showing the intersection of ubiquitination associated genes (URG) and DEGs. **(D, E)** GO **(D)** and KEGG **(E)** enrichment analysis of intersection difference URG.

The flowchart of the present study was shown in **Figure 1** (**Figure 1**). A total of 817 DEGs were identified from high and low BMD samples in the training cohort, consisting of 384 upregulated DEGs and 433 downregulated DEGs (**Figures 2A, B**). Upon overlapping these 817 DEGs with 1,367 URGs, 76 DE-URGs were successfully screened (**Figure 2C**). Enrichment analysis of the DE-URGs revealed 289 GO terms, including “protein polyubiquitination,” “protein deubiquitination,” and “proteasome-mediated ubiquitin-dependent protein catabolic process” (**Figure 2D**). These processes are part of the cellular mechanisms that regulate protein levels, activity, and function through dynamic modifications involving the addition or removal of ubiquitin or other small protein groups. In KEGG analysis, two significant pathways were identified: such as ubiquitin-mediated proteolysis (**Figure 2E**). These processes are crucial for protein quality control and homeostasis, with the ubiquitin-mediated proteolysis pathway playing a vital role in degrading improperly folded proteins recognized in the endoplasmic reticulum (ER), as well as those in the cytosol and nucleus. The ER ensures that only properly folded proteins progress through the secretory pathway, while misfolded proteins are either refolded or marked for destruction by the ubiquitin-proteasome system.

### 3.2 Combining RPS27A, PSMD4, UBE2E1, UCHL3, and RAD23A into a model of high diagnostic value

**Figure 3:**
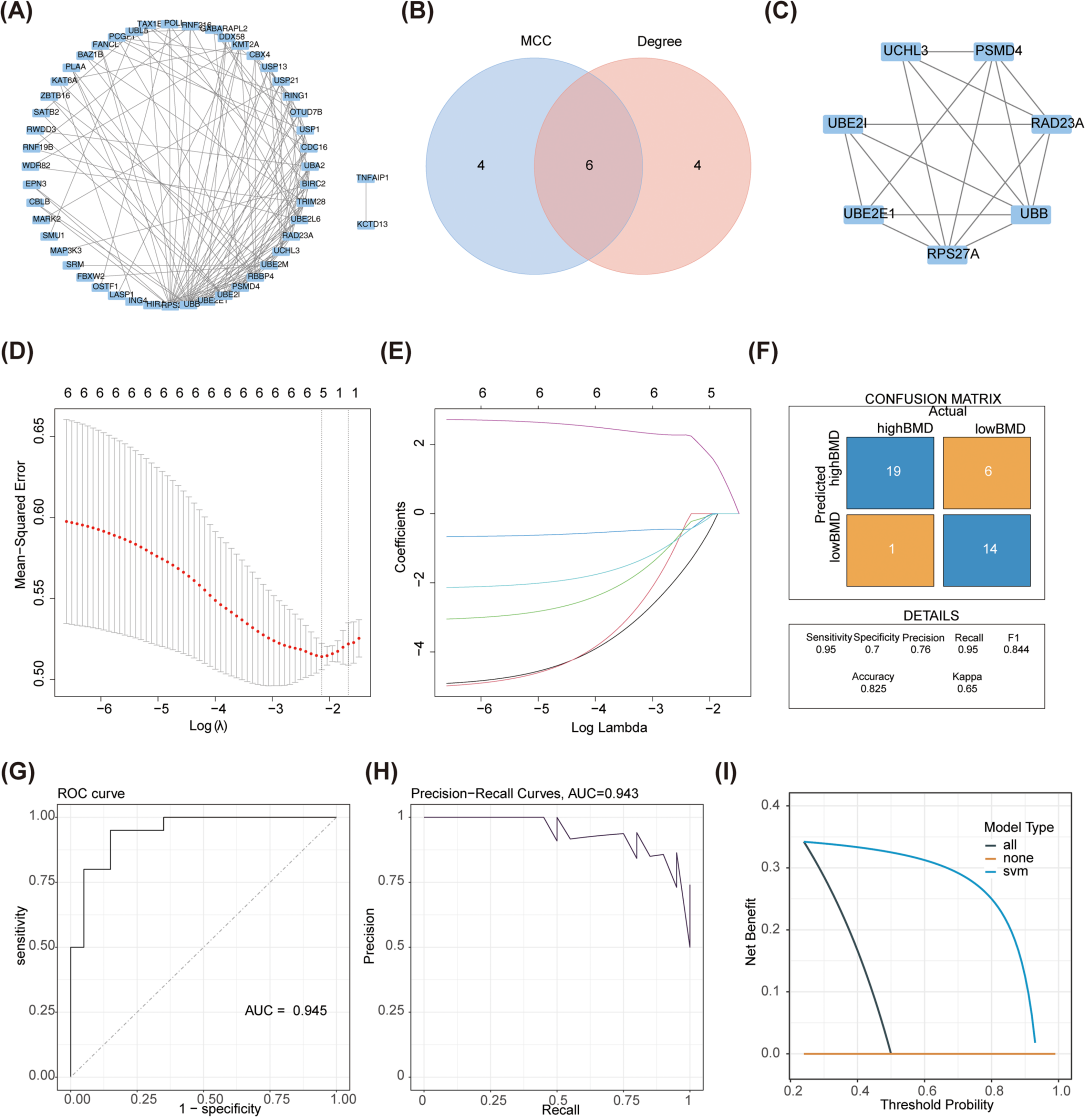
Construction of SVM model for candidate genes. **(A)** The PPI network map was constructed for the intersection difference URG genes. **(B)** The Venn diagram shows the intersection of MCC and Degree methods to obtain candidate genes. **(C)** PPI networks of candidate genes. **(D, E)** TMRGs related differential genes were screened by LASSO. **(D)** LASSO logic coefficient penalty diagram. The x-axis represents the logarithm of lambdas, and the y-axis represents the model error. **(E)** LASSO coefficient spectrum. The x-axis represents the logarithm of lambdas, and the y-axis represents the variable coefficients. **(F–H)**. **(F)** The confusion matrix. The X-axis represents actual values and the Y-axis represents predicted values. The top-left corner of the figure denotes True Positive (TP), the bottom-left corner denotes False Negative (FN), the top-right corner denotes False Positive (FP), and the bottom-right corner denotes True Negative (TN). Values on the diagonal indicate the count/proportion of correct predictions, while off-diagonal elements represent misclassifications. **(G)** ROC curve. The X-axis is the false positive rate (FPR) and the Y-axis is the true positive rate (TPR). AUC refers to the area under the curve, with a larger value indicating higher accuracy. **(H)** PR curve. The Y-axis is Precision and the X-axis is Recall, which is obtained by adjusting the threshold for distinguishing between Positive and Negative classes. **(I)** DCA. The X-axis represents Threshold Probability, and the Y-axis represents the net benefit rate after subtracting harms from benefits.

The 76 DE-URGs were incorporated into a PPI network, consisting of 50 nodes and 137 edges (**Figure 3A**). The top 10 genes identified by the MCC and Degree algorithms from the CytoHubba plugin were intersected, yielding six candidate genes: RPS27A, UBB, PSMD4, UBE2E1, UCHL3, and RAD23A (**Figures 3B, C**). These genes were further refined using a LASSO model, which minimized at lambda = 0.11806, leaving five genes: RPS27A, PSMD4, UBE2E1, UCHL3, and RAD23A (**Figures 3D, E**). An SVM model was subsequently constructed. The confusion matrix indicated a high number of correct predictions, with the AUC of 0.945, and the PR curve yielded an AUC of 0.943 (**Figures 3F–H**). These results demonstrate that the model is highly effective in distinguishing between high and low BMD samples. Decision curve analysis (DCA) further confirmed that the model provided a positive net benefit, highlighting its potential value in practical applications (**Figure 3I**).

### 3.3 Recognition of RPS27A and UBE2E1 as biomarkers

**Figure 4:**
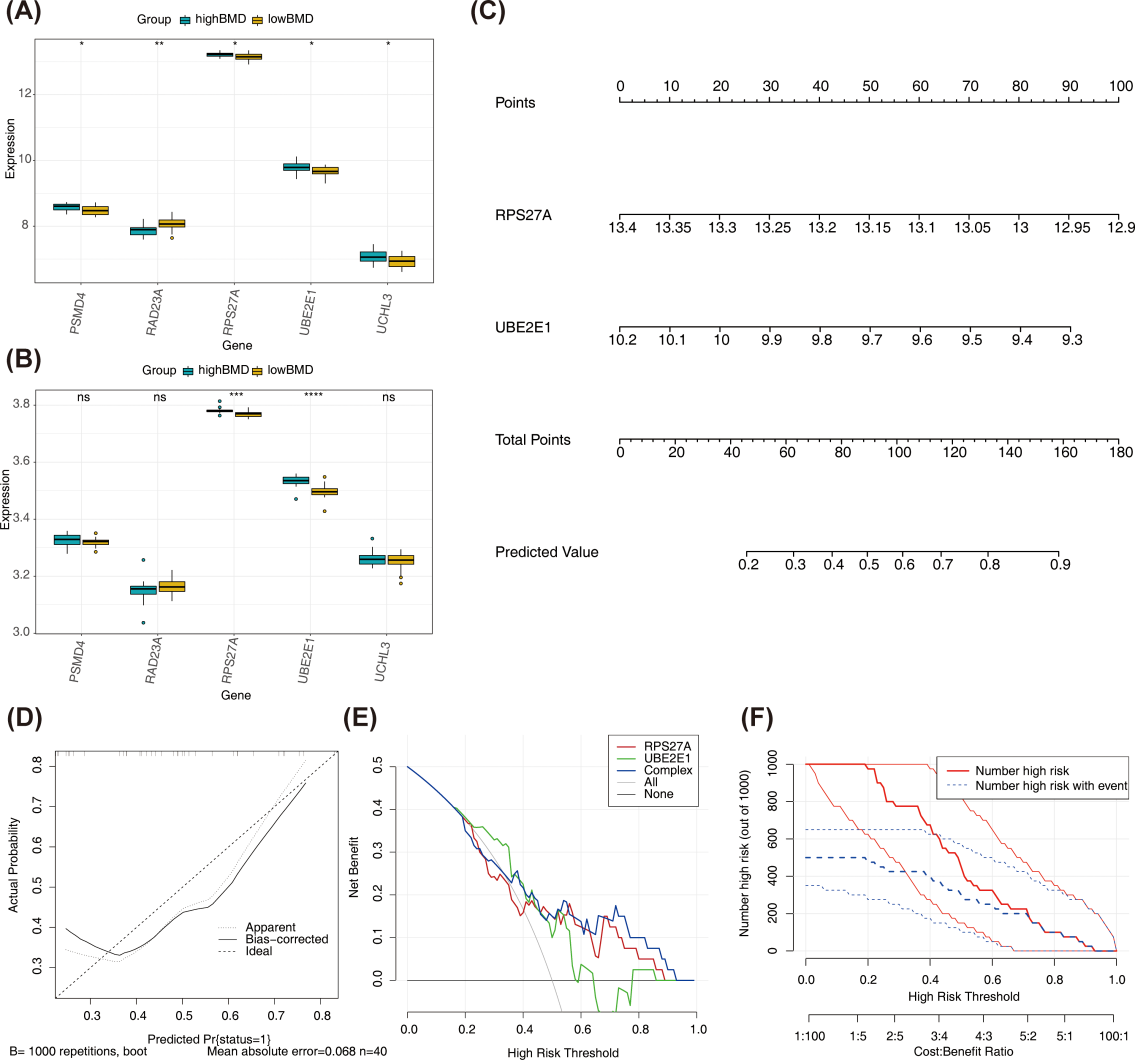
Identification of biomarkers **(A, B)** Expression levels of biomarkers in training sets **(A)** and validation sets **(B)** *p < 0.05, **p < 0.01, ***p < 0.001 were considered statistically significant **(C)** Nomogram of the SOP diagnostic model on the basis of 2 biomarkers. Each gene in the figure corresponds to a line segment marked with scale marks; the length of the segment reflects the magnitude of the factor’s contribution to the outcome event. The sum of individual scores gives the Total Point, and a higher Total Point indicates a better model. **(D)** The calibration was used to evaluate the diagnostic efficacy of the SOP diagnostic nomogram. **(E)** DCA illustrating the net binefit assessing the outcome. **(F)** The clinical impact curve showed the clinical effectiveness of the nomogram model. The red curve (Number of high risk) denotes the number of individuals classified as positive (high-risk) by the simple model at various threshold probabilities; the blue curve (Number of high risk with outcome) denotes the number of true positives at each threshold probability.

The expression levels of the five filtered genes were evaluated in both the training and validation cohorts. Notably, RPS27A and UBE2E1 exhibited consistent expression patterns across both datasets and showed significant differences (*P* < 0.05), with lower expression levels observed in samples with low BMD (**Figures 4A, B**). These findings led to the selection of RPS27A and UBE2E1 as biomarkers for this study, and they were subsequently used to construct a nomogram for predicting the risk of individual patients developing SOP and distinguishing the pathological characteristics of patient groups with varying BMD (**Figure 4C**). The calibration curve demonstrated an almost perfect alignment between the model’s estimated probabilities and the actual outcomes, with a concordance index (C-index) of 0.713 (**Figure 4D**). DCA indicated that the model’s net benefit decreased as the threshold probability increased; however, the model performed well across the range of threshold probabilities, except for very low threshold values (**Figure 4E**). The clinical impact curve highlighted the clinical utility of this predictive model (**Figure 4F**). By analyzing the expression of URGs in SOP patients, the nomogram model not only provided a deeper understanding of the disease’s molecular mechanisms but also showed promise for developing innovative therapeutic strategies.

### 3.4 Correlations between biomarkers and biological functions: insights into signaling pathways and health implications

**Figure 5:**
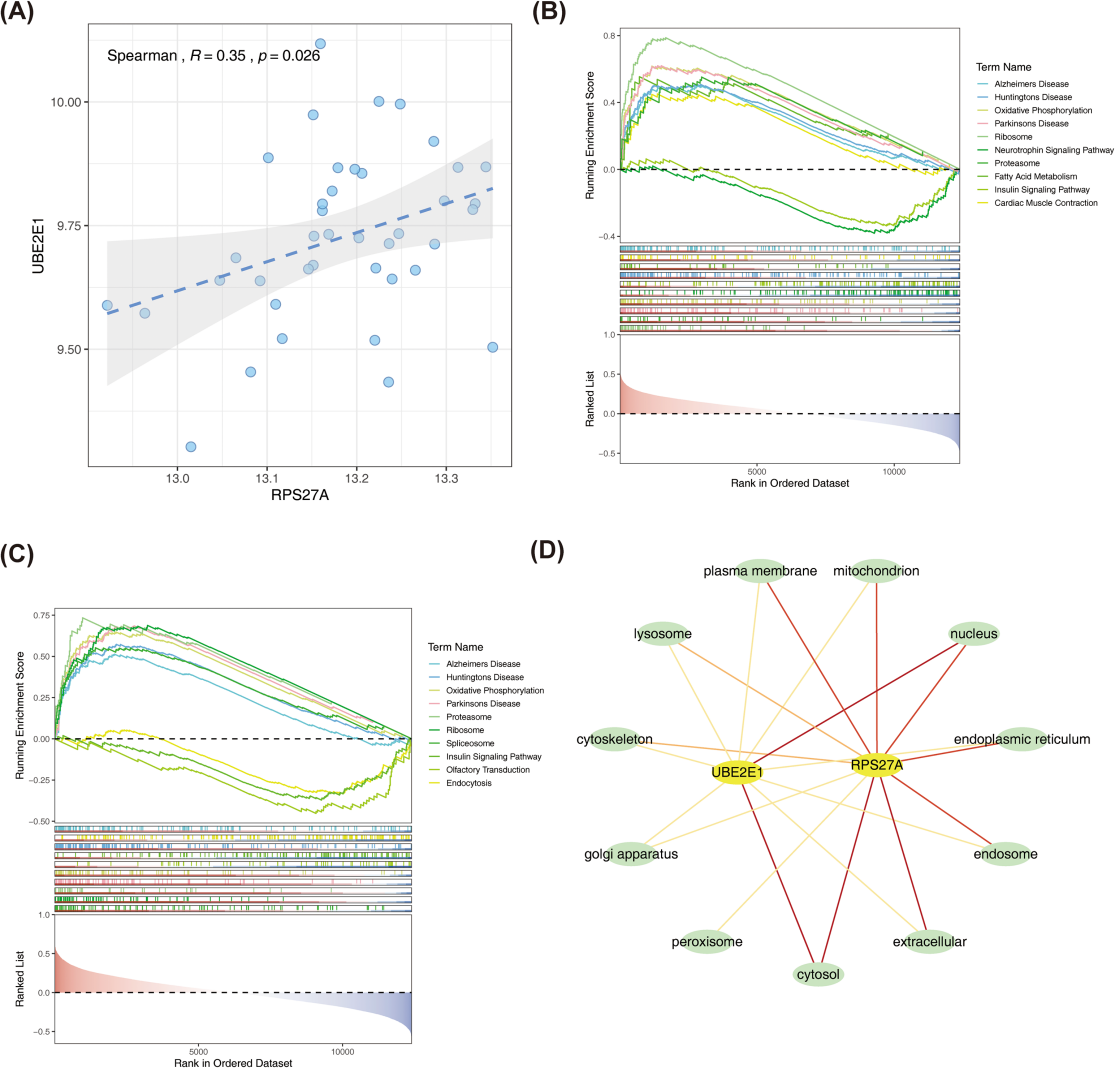
Correlation and biological functions of RPS27A and UBE2E1 **(A)** Correlation analysis showed a moderate positive correlation between RPS27A and UBE2E1 **(B, C)** GSEA shows the related pathways and functions enriched by RPS27A **(B)** and UBE2E1 (CD. Subcellular localization of RPS27A and UBE2E1. Yellow indicates content related to RPS27A and UBE2E1, and green indicates subcellular localization.

A moderate positive correlation was observed between the two biomarkers, RPS27A and UBE2E1 (cor = 0.35, *P* = 0.026) (**Figure 5A**). GSEA revealed that both RPS27A and UBE2E1 were enriched in pathways related to “Oxidative phosphorylation,” “Proteasome,” “Ribosome,” and “Insulin signaling pathway” (**Figures 5B, C**). These pathways are involved in neurodegenerative diseases, essential cellular functions (such as energy production, protein synthesis, and degradation), and critical intracellular signaling mechanisms, suggesting that these biomarkers could have significant mechanistic implications for SOP patients across varying BMD levels.

Additionally, a subcellular-biomarker network constructed using GeneCards showed that RPS27A is localized to the cytosol and extracellular space (**Figure 5D**). This localization suggests that RPS27A may play a role in fundamental cytoplasmic functions, such as protein synthesis through its interaction with ribosomes, and may also be involved in extracellular processes, such as protein release during cell damage. UBE2E1, on the other hand, was found in both the cytosol and the nucleus, indicating its potential involvement in intracellular transport, nucleo-cytoplasmic exchange, and its possible direct influence on gene expression and the maintenance of genomic stability within the nucleus.

### 3.5 Biomarkers as representatives of immune microenvironment status in SOP patients

**Figure 6:**
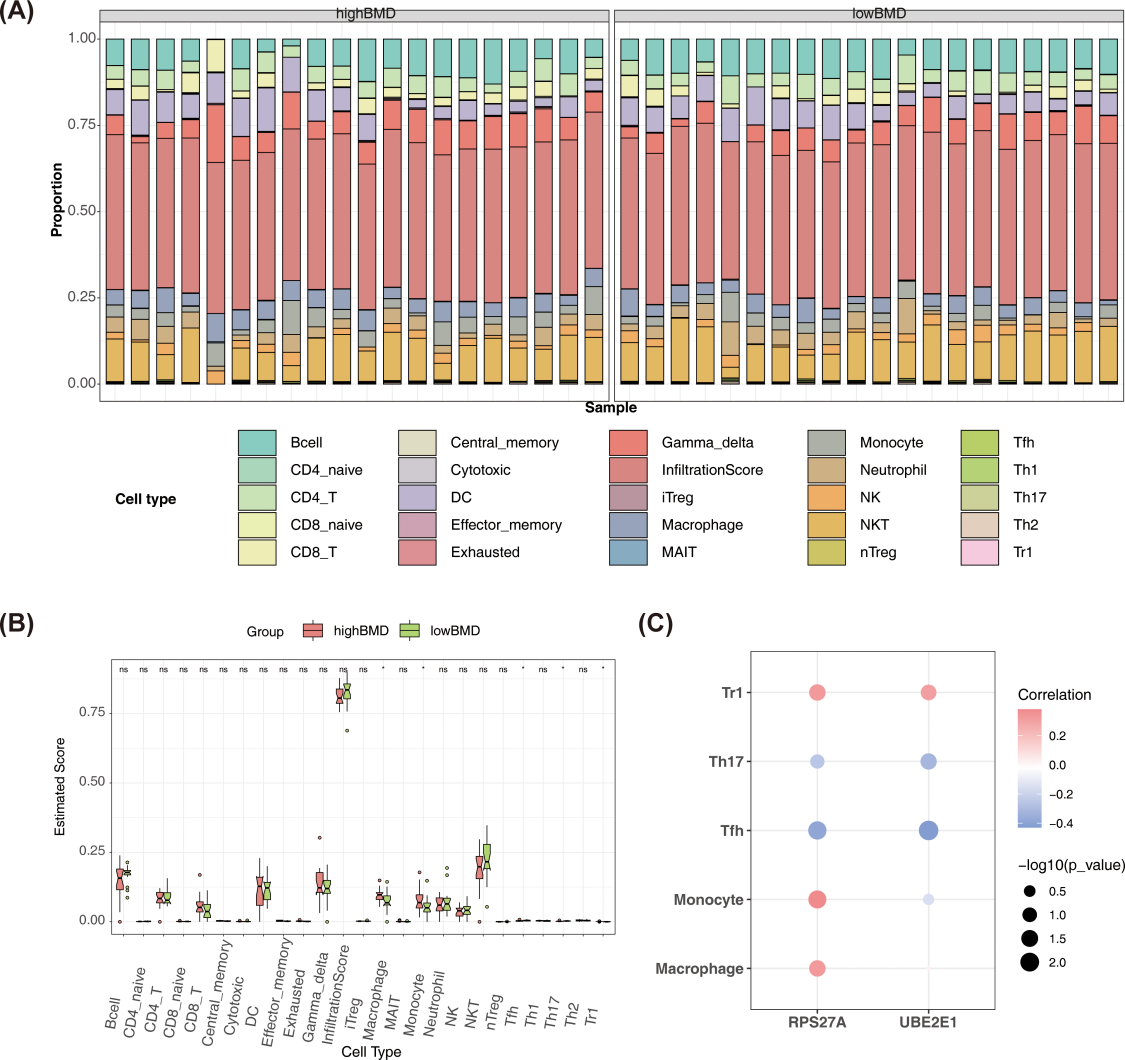
Immunoinfiltration analysis of RPS27A and UBE2E1. **(A, B)** Stack **(A)** and box **(B)** showed that there were five types of immune cells with significant differences between the disease and control groups. The X-axis represents different samples, and the Y-axis represents the proportion of immune cells. *p < 0.05 was considered statistically significant. **(C)** Correlation analysis of RPS27A and UBE2E1 with five kinds of immune cells. The X-axis represents biomarkers, and the Y-axis represents differentially expressed immune cells.

To explore the correlation between biomarkers and the immune microenvironment in SOP patients with different BMD levels, an immune infiltration analysis was performed. The assessment of immune cell infiltration across 25 immune cell types in patients with high and low BMD revealed significant differences in five cell types (*P* < 0.05) (**Figures 6A, B**). Macrophages, monocytes, and T regulatory 1 cells (Tr1) exhibited higher abundance in high BMD samples (*P* < 0.05), while T helper 17 cells (Th17) and T follicular helper cells (Tfh) were more prevalent in low BMD samples (*P* < 0.05), potentially contributing to the pathogenesis of OP. Correlation analysis demonstrated that RPS27A exhibited moderate correlations with all immune cells except Th17 (|cor| > 0.3), while UBE2E1 showed moderate correlations with Tfh, Th17, and Tr1 (|cor| > 0.3) (**Figure 6C**). These results suggest that RPS27A and UBE2E1 may regulate specific immune cell types in the microenvironment. In particular, the strongest positive correlation was observed between Tfh and UBE2E1 (cor = 0.43, *P* < 0.01), suggesting that UBE2E1 may influence autoimmune diseases and inflammatory responses. Additionally, monocytes and RPS27A showed the strongest positive correlation (cor = 0.38, *P* < 0.05), indicating that RPS27A might modulate or respond to immune processes driven by monocytes.

### 3.6 Speculating the potential molecular mechanisms of biomarkers

**Figure 7:**
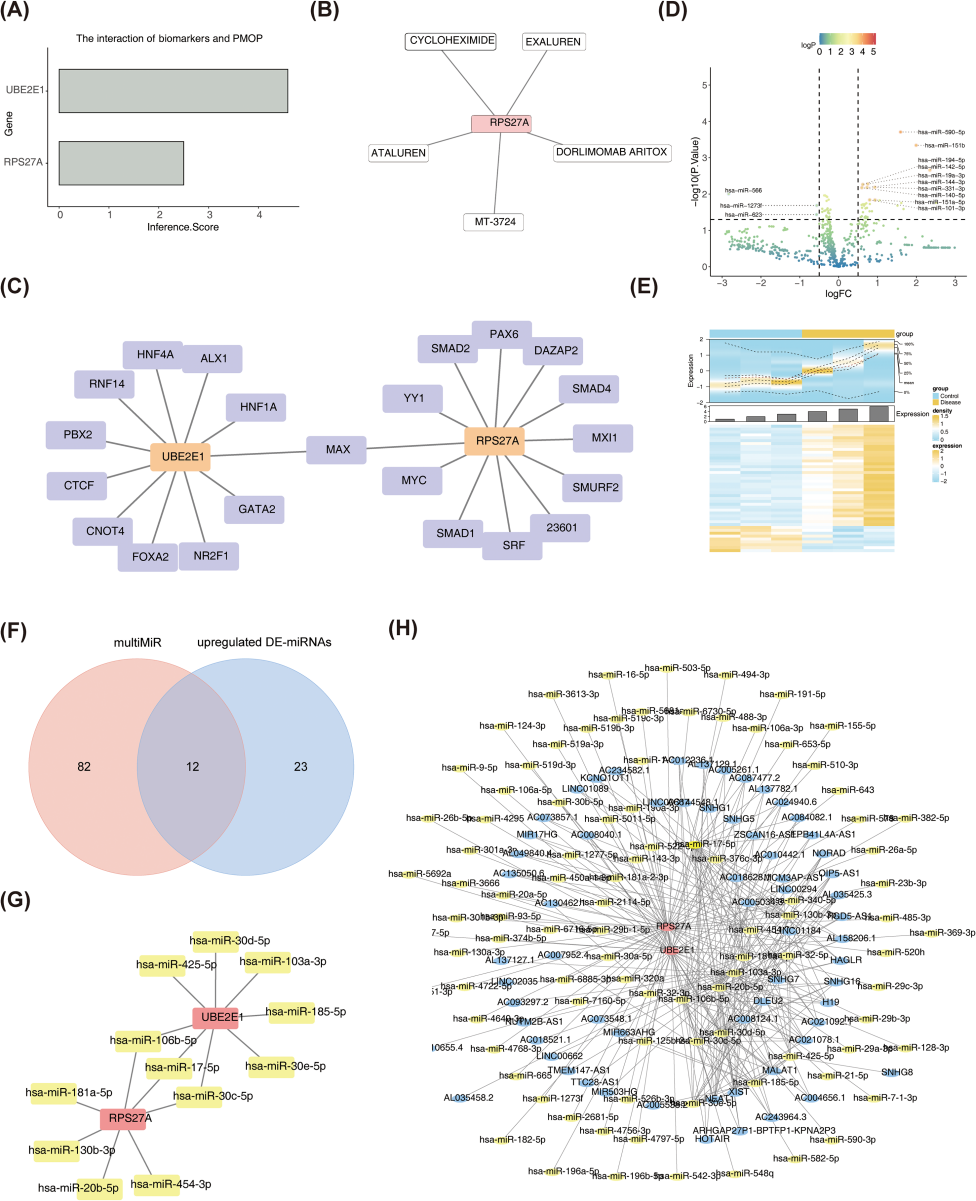
Potential mechanism prediction and drug prediction analysis of RPS27A and UBE2E1 in SOP occurrence **(A)** The CTD database evaluates the relationship between RPS27A and SOP and the relationship between UBE2E1 and SOP. **(B)** Potential therapeutic agents for RPS27A and UBE2E1. **(C)** Network diagram of RPS27A and UBE2E1 interacting with transcription factors. Orange dots represent mRNAs, and purple dots represent transcription factors. **(D)** Volcanic maps of miRNA differential expression in disease and control groups. Divided by reference lines, the genes in the upper right corner are upregulated miRNA, the genes in the upper left corner are downregulated miRNA, and the rest are miRNA with no significant statistical difference. **(E)** Heat maps of miRNA differential expression in disease and control groups.Yellow indicates highly expressed miRNA, and blue indicates lowly expressed miRNA. **(F)** The Venn diagram indicated that the predicted miRNA of RPS27A and UBE2E1 were intersected with significantly differentially expressed mRNA. **(G)** The miRNA network diagram of RPS27A and UBE2E1 was adjusted jointly. Red dots represent mRNAs, and yellow dots represent miRNAs. **(H)** Construction of ceRNA network diagram for RPS27A and UBE2E1. Blue dots represent lncRNAs, yellow dots represent miRNAs, and red dots represent mRNAs.

The association between biomarkers and SOP was further explored using the CTD, which revealed a strong correlation between UBE2E1 and SOP, with an inference score greater than 4. In contrast, RPS27A exhibited a moderate correlation with SOP, with an inference score over 2 (**Figure 7A**). Additionally, potential drugs targeting these biomarkers were identified using the DGIdb database, with RPS27A interacting with drugs such as EXALUREN, CYCLOHEXIMIDE, ATALUREN, MT-3724, and DORLIMOMAB ARITOX (**Figure 7B**).

To identify potential regulatory TFs for the biomarkers, a TF-mRNA network was constructed using the miRNet database, resulting in a network comprising 23 edges and 24 nodes. MAX was identified as a regulator of both genes (**Figure 7C**). Differential analysis of miRNA expression between SOP patients and control subjects revealed 44 DE-miRNAs, with 35 upregulated and 9 downregulated (**Figures 7D, E**). Using the multiMiR package, 195 miRNAs were predicted to target RPS27A and 51 miRNAs to target UBE2E1. By intersecting these miRNAs with those showing inverse expression to the mRNAs, a set of 12 miRNAs was identified, leading to the construction of a miRNA-mRNA regulatory network (**Figure 7F**). Among these, three miRNAs, including hsa-miR-106b-5p, were found to co-regulate both RPS27A and UBE2E1 (**Figure 7G**). Further investigation in the starBase database identified corresponding lncRNAs for these 12 miRNAs, resulting in the construction of a complex lncRNA-miRNA-mRNA network with 88 nodes and 279 edges, which included two mRNAs, 12 miRNAs, and 64 lncRNAs (**Figure 7H**). This network revealed multiple interactions, such as the regulation of UBE2E1 by lncRNAs like AC005538.2, XIST, NEAT1, MALAT1, and AC130462.1 *via* hsa-miR-185-5p.

### 3.7 Successful establishment of the SOP model and verification of biomarkers

**Figure 8:**
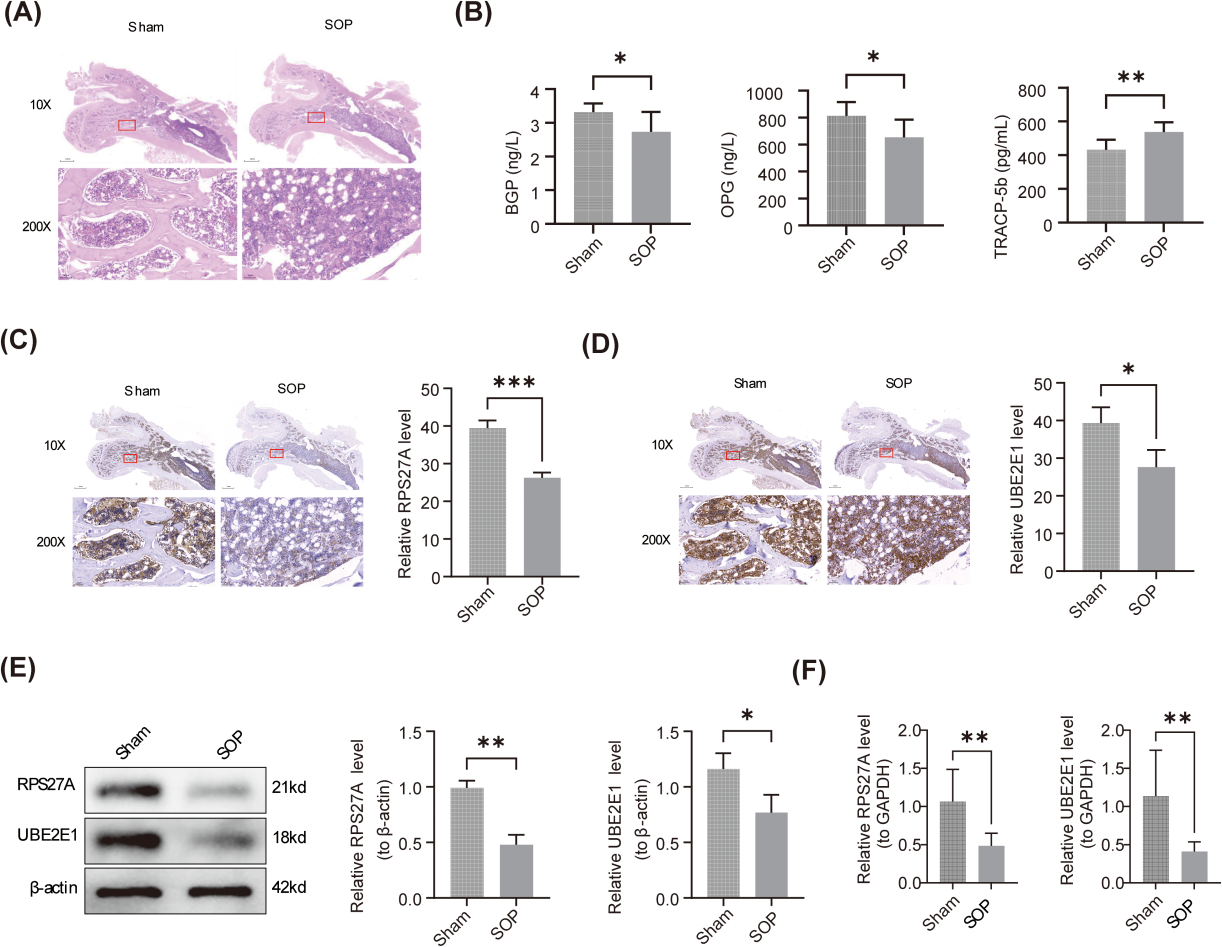
Validation of biomarkers in a rat model of SOP **(A)** HE staining of femur tissues in control group and disease group.10× Scale bars, 1mm,200× Scale bars, 50μm. **(B)** Serum concentrations of BGP, OPG and TRACP5b in control group and disease group were detected by ELISA Representative images from immunohistochemical staining of RPS27A **(C)** and UBE2E1 **(D)** in the Femur tissues. 10× Scale bars, 1mm,200× Scale bars, 50μm. **(E)** Detection of RPS27A and UBE2E1 in femur tissues from control group and disease group by western blot. Total GAPDH was used as a loading control. Data are mean ± s.d. (n=3),*p<0.05, by Student’s t-test. **(F)** RPS27A and UBE2E1 mRNA in femur tissue of rats in control group and disease group were measured by RT-qPCR. Data are mean ± s.d. (n=3). *p<0.05, ** p < 0.01, and *** p < 0.001 were considered statistically significant by Student’s t-test.

Vaginal smear analysis revealed that rats in the Sham group remained in the estrus stage, while those in the SOP group predominantly exhibited white blood cells, indicating successful induction of senescence (**Supplementary Figure 1A**). No significant differences in the gross appearance of femoral tissues were observed between the Sham and SOP groups (**Supplementary Figure 1B**). Additionally, the body weight of SD rats increased over time in both groups (**Supplementary Figure 1C**). H&E staining showed that the bone tissue structure in the Sham group was dense and regular, with tightly arranged and continuous trabeculae. In contrast, the SOP group displayed sparse trabecular structure, increased gaps between trabeculae, and numerous irregular cavity-like structures, confirming successful model establishment (**Figure 8A**). ELISA results revealed that, compared to the Sham group, BGP and OPG levels were significantly reduced in the SOP group (*P* < 0.05), while TRACP5b levels were elevated (*P* < 0.01) (**Figure 8B**). IHC analysis indicated a decrease in the expression of RPS27A and UBE2E1 in the SOP group compared to the Sham group (**Figures 8C, D**). WB analysis confirmed that expression levels of both biomarkers were lower in the SOP group than in the Sham group (*P* < 0.05) (**Figure 8E**). Notably, RT-qPCR results were consistent with those of WB, showing significantly lower expression of the biomarkers in the SOP group compared to the Sham group (*P* < 0.01) (**Figure 8F**).

## 4 Discussion

SOP is a prevalent metabolic bone disease, particularly in postmenopausal elderly women (35). Age-related bone loss is primarily caused by an imbalance in bone remodeling (36). It has been reported that the ubiquitin-proteasome pathway plays a pivotal role in bone remodeling (15). However, the biomarkers and molecular mechanisms of this pathway in elderly patients with OP remain unclear. This study mined the GEO database to analyze DEGs and constructed a PPI network for DEGs related to ubiquitination. By combining the MCC algorithm and Degree algorithm in the Cytoscape plug-in with LASSO analysis, five key genes were identified: RPS27A, PSMD4, UBE2E1, UCHL3, and RAD23A. Subsequently, an SVM model was constructed based on these five genes to assess the diagnostic accuracy of the model in distinguishing between the disease and control groups. Further analysis revealed that RPS27A and UBE2E1 exhibited significant differences and consistent expression trends in both the training and validation sets. This multi-dimensional screening approach evaluates gene importance from both local and global perspectives, reducing the number of candidate biomarkers and improving the accuracy and reliability of the screening process (37–40). This study provides a theoretical foundation and data support for the prevention and treatment of OP in elderly women.

RPS27A (Ribosomal Protein S27a) and UBE2E1 (Ubiquitin Conjugating Enzyme E2E1) are two biomarkers involved in cellular function and various biological processes. The RPS27A gene encodes a component of the 40S ribosomal subunit, playing a pivotal role in protein synthesis (41). The encoded protein is also part of the ubiquitin-proteasome pathway, functioning as a fusion protein with an N-terminal ubiquitin tag (42) that facilitates the degradation of target proteins (42). Previous studies have shown that RPS27A expression is significantly down-regulated in postmenopausal women with OP (43), and recent research has also found down-regulation of RPS27A expression in elderly patients with OP (44), consistent with our findings. Compared with previous studies, this research incorporates two key influencing factors of aging and menopause, focusing on postmenopausal elderly women as the specific population. Through multiple-step screening, RPS27A is identified as a biomarker with diagnostic value for osteoporosis in this specific group. Moreover, the support vector machine disease prediction model constructed further validates this conclusion. Additionally, this research also reveals the association between RPS27A and the immune microenvironment, enriching the understanding of the role of this gene in the pathogenesis of osteoporosis.The UBE2E1 gene encodes a member of the ubiquitin ligase family, playing a critical role in the ubiquitination pathway by attaching ubiquitin tags to proteins, marking them for proteasomal degradation (45). Some studies have suggested that UBE2E1 may be involved in neurodegenerative diseases such as Alzheimer’s disease and Parkinson’s disease (46), and elevated levels of UBE2E1 may also be associated with inflammatory and autoimmune diseases (47). To date, there have been no literature reports indicating a correlation between it and osteoporosis. However, this study is the first to discover the correlation between the two and link it to the disease risk. Some researchers have found that this gene can regulate the classical WNT pathway (48), and the Wnt/β-catenin signaling pathway is considered a key pathway for osteoblast differentiation and has become an important target for the treatment of osteoporosis (49). UBE2E1 may be involved in the occurrence and development of senile osteoporosis through the classical Wnt pathway. This finding has not been reported in the literature yet and requires further verification. Overall, RPS27A and UBE2E1 are two newly identified key ubiquitination genes that hold promise not only as biomarkers for OP but also as potential therapeutic targets. Notably, this study developed a nomogram that integrates these two diagnostic markers with high AUC values and excellent calibration. This nomogram demonstrated outstanding accuracy and reliability in diagnosing OP and is expected to be clinically applied to facilitate early diagnosis of SOP.

GSEA was performed to further explore the potential functions and pathways associated with these two biomarkers in OP. The analysis revealed co-enrichment in seven pathways: “Alzheimer’s disease,” “Huntington’s disease,” “Oxidative phosphorylation,” “Parkinson’s disease,” “Proteasome,” “Ribosome,” and “Insulin signaling pathway.” The insulin signaling pathway is a critical, versatile pathway with multiple anabolic functions beyond glucose homeostasis (50). It regulates essential aspects of organismal health, such as growth, longevity, metabolism, and reproduction (50). Activation of the insulin signaling pathway can enhance osteoblast differentiation and promote bone formation (51, 52), while dysfunction in this pathway leads to bone formation disorders and OP (53). Furthermore, several studies have suggested a link between the development of OP in the elderly and ribosome-related genes and pathways (44, 54). The proteasome pathway recognizes proteins tagged with ubiquitin and degrades them through hydrolysis. Our study further confirms that the ubiquitin-proteasome pathway plays a significant role in OP, particularly in elderly patients. The ubiquitination of proteins generally occurs in the cytoplasm. The subcellular localization analysis of the biomarkers revealed that both RPS27A and UBE2E1 are expressed in the cytoplasm, further supporting the idea that key steps in regulating OP development may occur in the cytoplasm.

During the onset and progression of OP, the immune system plays a pivotal role in bone metabolism. Chronic low-grade inflammation is now recognized as a significant contributor to OP, particularly in postmenopausal women and the elderly (55). In the present study, immune infiltration analysis revealed significant differences in the infiltration levels of macrophages, monocytes, Tfh, Th17, and Tr1 cells. Th17 cells, a subset of T cells, promote inflammation by directly expressing RANKL, which facilitates the interaction between receptor activator of nuclear factor-κB ligand (RANKL)and receptor activator of nuclear factor-κB (RANK). These cells also secrete inflammatory cytokines such as IL-17, TNF-α, and IL-6, which stimulate osteoclasts (OCs) and enhance inflammatory infiltration. This process increases the expression of NF-κB, which further upregulates RANKL expression, promoting OC maturation and differentiation (56). However, the role of Th17 cells in osteoblasts remains controversial, with studies showing both supportive and inhibitory effects on bone metabolism (8). Increased IL-17 levels in postmenopausal OP patients correlate with a higher frequency of Th17 cells (57). Aging also leads to an increased Th17/Treg ratio, which may induce a low-activation state of the immune system, promote the production of inflammatory factors, and contribute to bone loss (58). Our study found that Th17 cells have a high infiltration abundance in elderly postmenopausal OP patients, and UBE2E1 is significantly negatively correlated with Th17, suggesting that UBE2E1 may influence the internal environment of OP patients through Th17 cells.

Tfh, a subset of CD4+ Th cells, play a critical role in B cell activation, proliferation, and differentiation (59). These processes predominantly occur in B cell follicles within secondary lymphoid organs and are essential for mounting effective antibody responses (59). Tfh are also involved in various conditions, including cancer, allergies, autoimmune diseases, and infections (59). Although Tfh have widespread effects, their association with OP has not been well documented. However, Tfh may be involved in bone metabolism disorders caused by immune-related diseases such as rheumatoid arthritis (60), systemic lupus erythematosus (61), and schistosomiasis (62). In our study, both RPS27A and UBE2E1 were significantly negatively correlated with Tfh, and their expression levels were high in OP patients, indicating that RPS27A may also serve as an immune factor in the pathogenesis of SOP. These findings offer new insights for future research, guiding researchers to explore the specific roles of these genes in immune responses and related diseases. Additionally, they may contribute to the development of novel therapeutic strategies for OP.

Through our research combined with previous literature reports, we discovered that UBE2E1 can work in synergy with the E3 ligase RNF34 to ubiquitinate ciliary protein MKS1. This process can precisely regulate the level of the core transcription factor β-catenin in the classical Wnt signaling pathway (48), and the abnormality of β-catenin can activate Th17 differentiation and inhibit Treg cells (15), thereby disrupting the bone immune balance and leading to increased bone resorption. Similarly, in the study of RPS27A, it was found that RPS27A can promote M2 polarization of macrophages. Although this study mainly focused on cancer rather than bone diseases, it revealed the regulatory role of RPS27A on macrophage polarization (63), and macrophage polarization plays a key role in bone remodeling (64, 65). Therefore, we speculate that as a ribosomal protein and ubiquitin precursor, RPS27A may participate in the pathogenesis of osteoporosis by influencing macrophage polarization through releasing certain signals. We believe that the ubiquitination system is the core regulator of immune cell activation and inflammatory factor signal transduction, and its participation in the chronic inflammatory response in the osteoporosis microenvironment may lead to the disruption of bone homeostasis and is an important link in the pathogenesis of osteoporosis.

This study predicted the upstream regulators of RPS27A and UBE2E1 through molecular regulatory network analysis, identifying hsa-miR-106b-5p, hsa-miR-17-5p, and hsa-miR-30c-5p as key regulators of these genes. Notably, hsa-miR-106b-5p has been shown to influence bone formation and OP through the regulation of TCF4 (66). It is hypothesized that RPS27A and UBE2E1 are regulated by the upstream miRNA hsa-miR-106b-5p, contributing to the promotion of OP. This insight could offer new perspectives and potential drug targets for OP research. Finally, we established the SOP model and verified the expression levels of RPS27A and UBE2E1 at both the protein and mRNA levels. It was found that the protein expressions of RPS27A and UBE2E1 in the SOP group were lower than those in the control group. The immunohistochemical results were also in line with expectations, and this was consistent with the expression at the transcriptional level. The above results indicate that RPS27A and UBE2E1 may be involved in the pathological process of SOP through the ubiquitin-mediated pathway. This study provides new insights into the role of the ubiquitin-mediated pathway and supports the potential of these two biomarkers as biomarkers for diagnosing and treating SOP.

In conclusion, by analyzing elderly postmenopausal OP samples and healthy samples from the GEO high-throughput database and applying bioinformatics and machine learning techniques, this study identified RPS27A and UBE2E1 as biomarkers in the ubiquitin-proteasome pathway involved in the development of OP in the elderly. The findings offer valuable insights into the molecular mechanisms of ubiquitination in SOP. However, these conclusions are based on health data analysis, and additional clinical sample data are required for further validation. Future research will continue to explore the role of these mechanisms and conduct experimental studies to confirm their scientific accuracy.

## Data Availability

The original contributions presented in the study are included in the article/**Supplementary Material**. Further inquiries can be directed to the corresponding author.
