# Supplementary material for: Identification and validation of ubiquitination-associated genes of senile osteoporosis based on bioinformatics analysis
Source: Front Immunol. 2025 Dec 12;16:1629276. doi: 10.3389/fimmu.2025.1629276 (PMC12740899; doi:10.3389/fimmu.2025.1629276)
Supplement: Supplementary file 1 [file DataSheet1.docx]

Supplementary Material

Supplementary Figure


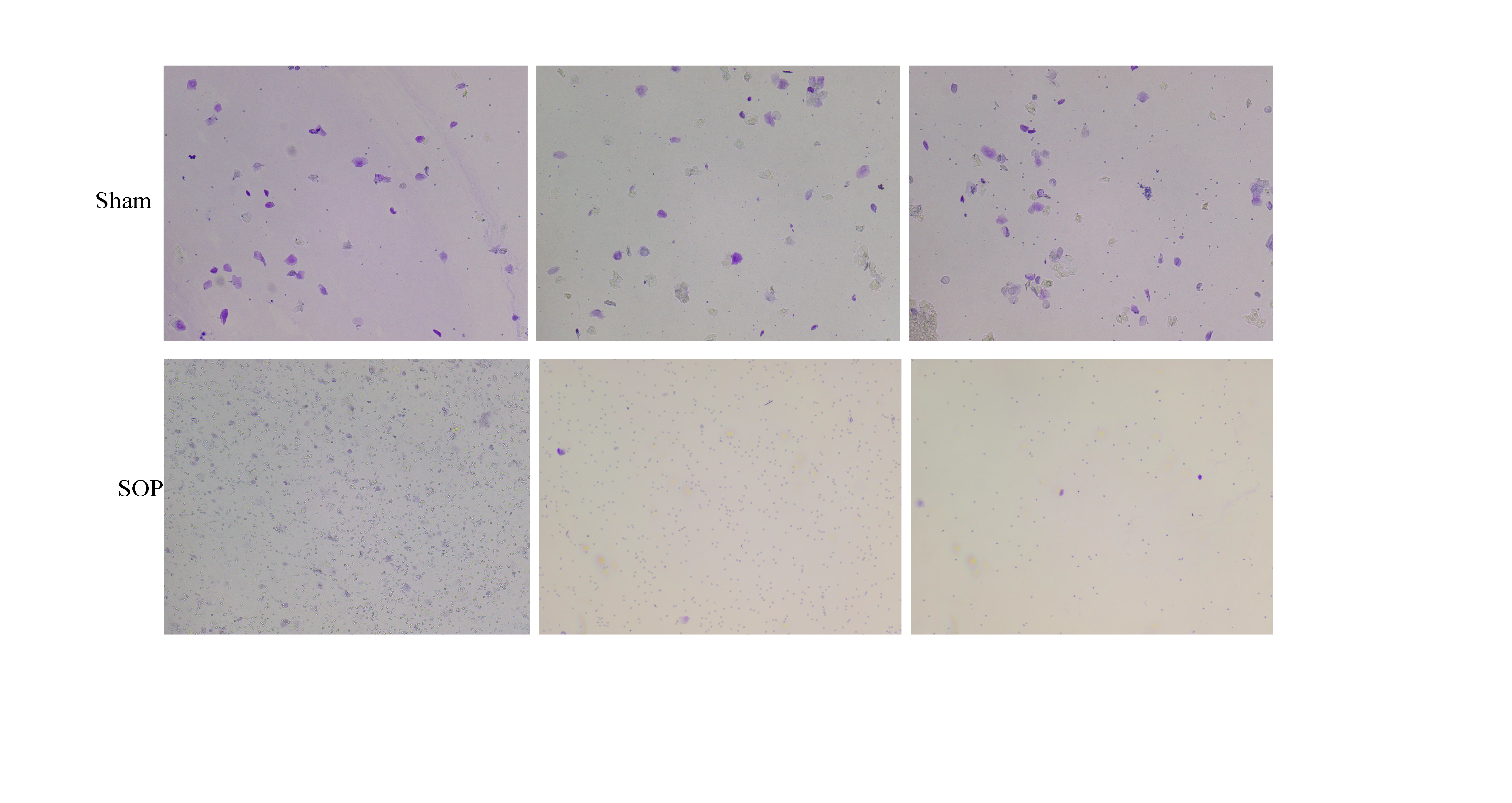


**Supplementary Figure 1A Comparison of vaginal images between the sham group and SOP group**


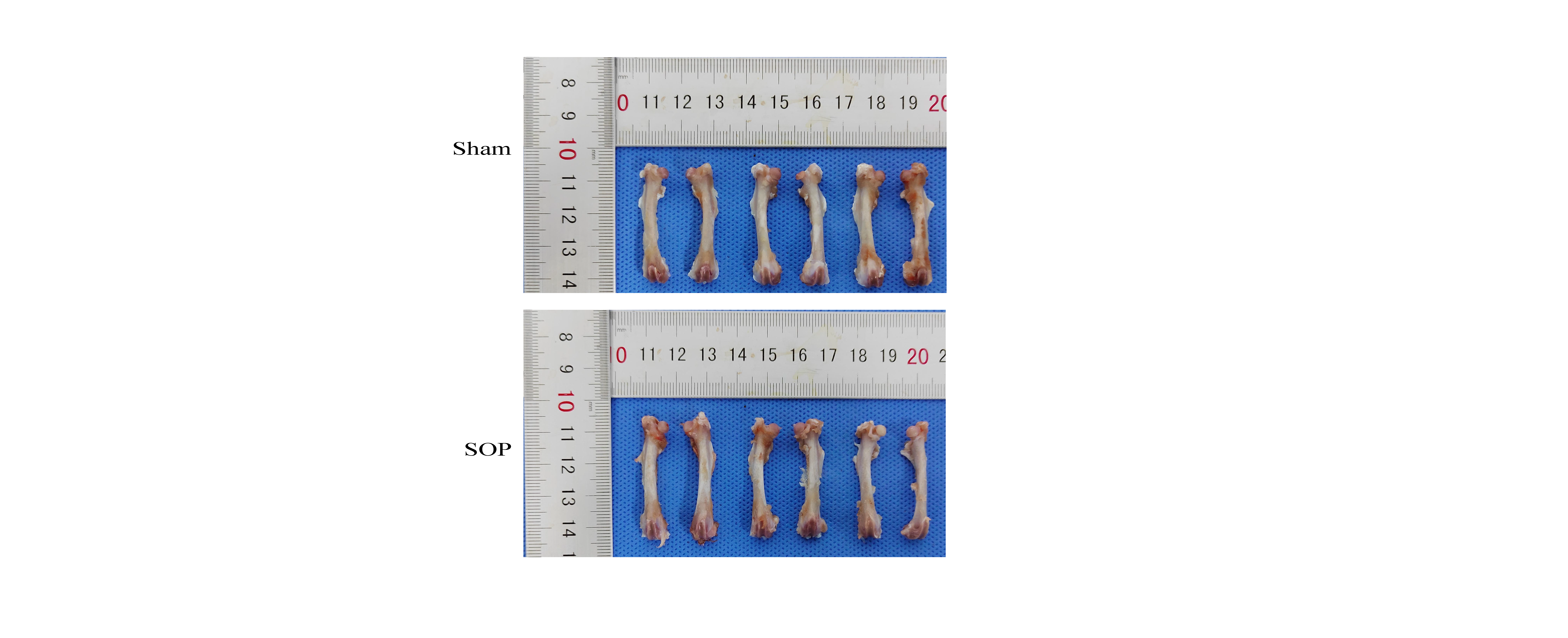

**Supplementary Figure 1B Comparison of femoral appearance between the sham group and the SOP group**


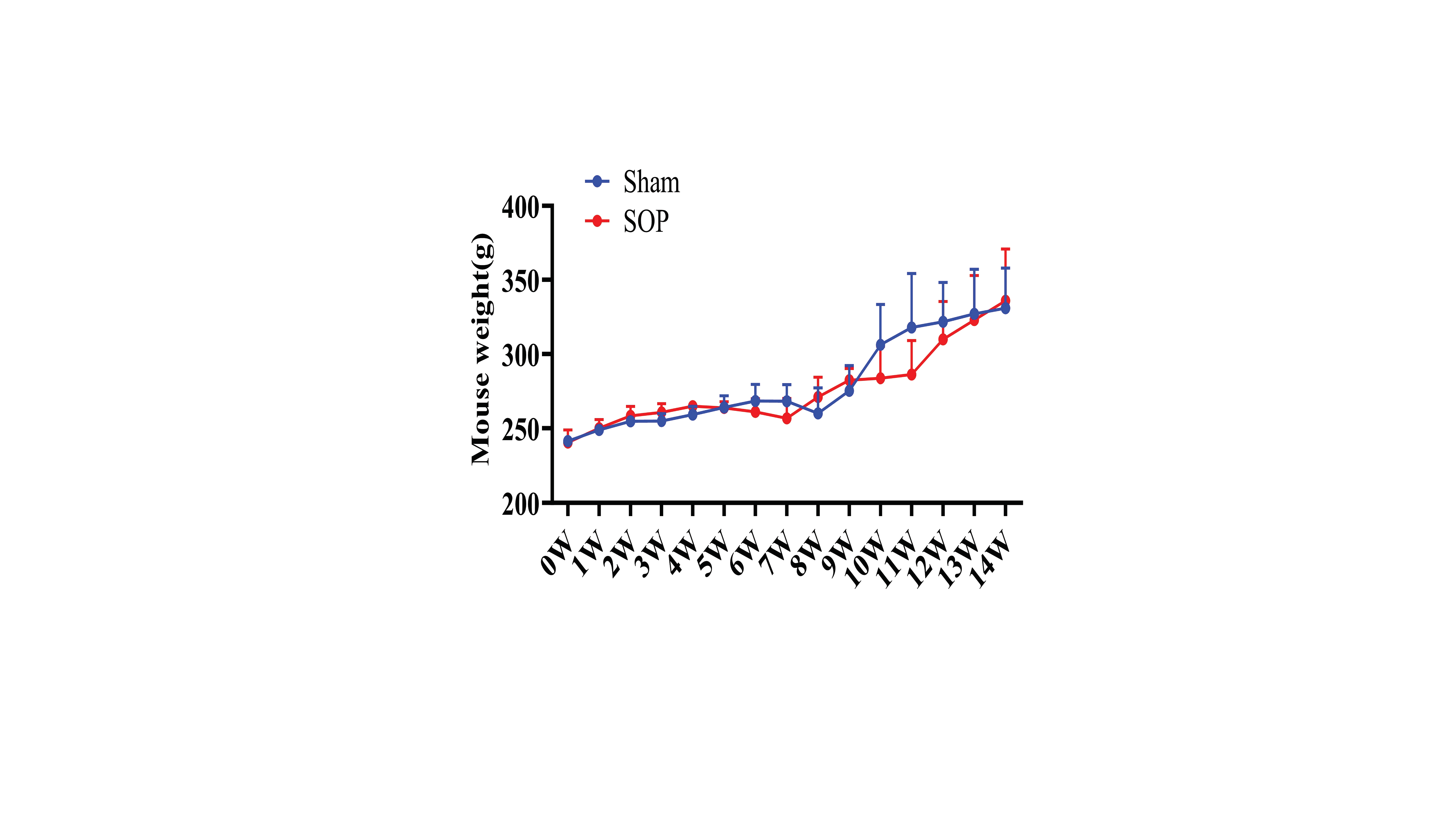

**Supplementary Figure 1C Weight changes of rats in the sham group and the SOP group**


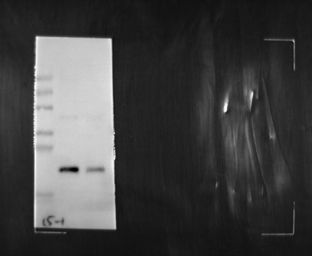


**Supplementary Figure 2A The full scan of the entire original gel(s) of RPS27A**


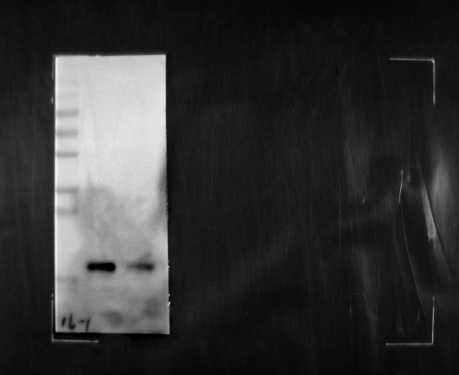


**Supplementary Figure 2B The full scan of the entire original gel(s) of UBE2E1**


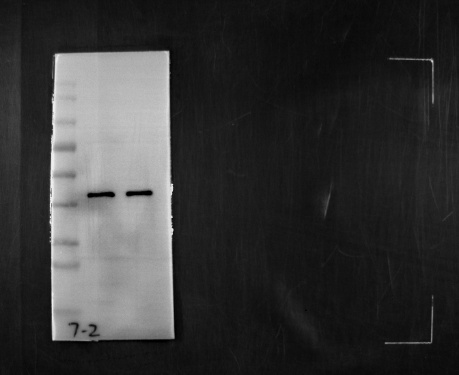


**Supplementary Figure 2C The full scan of the entire original gel(s) of β-actin**

**Supplementary Table**

**Supplementary table 1 WB antibody information and dilution ratio**

| Type | Manufacturers | Art.No.(article number) | Dilution ratio |
| --- | --- | --- | --- |
| RPS27A | Proteintech | 14946-1-AP | 0.736111111 |
| UBE2E1 | Affinity | DF14541 | 1.430555556 |
| β-actin | Proteintech | 66009-1-Ig | 1:25000 |
| HRP labeled goat anti-rabbit IgG | Servicebio | GB23303 | 2.125 |
| HRP labeled goat anti-mouse IgG | Servicebio | GB23301 | 3.513888889 |

**Supplementary table 2 qPCR reaction system and primer sequences**

| **Component** | | **Volume** | | |
| --- | --- | --- | --- | --- |
| cDNA | | 3μL | | |
| 2xUniversal Blue SYBR Green qPCR Master Mix | | 5μL | | |
| Forward primer (10µM) | | 1μL | | |
| Reverse primer (10µM) | | 1μL | | |
|  | **Temperature** | | **Time** |  |
| Pre - denaturation | 95℃ | | 1min |  |
| Denaturation | 95℃ | | 20s |  |
| Annealing | 55℃ | | 20s |  |
| Extension | 72℃ | | 30s |  |

| **primer** | **sequence** |
| --- | --- |
| RPS27A F | ACTGGTGTGGTCGGGTCTAAT |
| RPS27A R | CCACCACGAAGTCTCAGCAC |
| UBE2E1 F | ACACAAAGAGAGGGGCCGTT |
| UBE2E1 R | GCTGGTTGGAGGACGAAGAT |
| GAPDH F | GGCCGGAGACGAATGGAAATTA |
| GAPDH R | CCAAATCCGTTCACACCGAC |
